# Metabolomic and Transcriptomic Analyses Reveal the Potential Mechanisms of Dynamic Ovarian Development in Goats during Sexual Maturation

**DOI:** 10.3390/ijms25189898

**Published:** 2024-09-13

**Authors:** Yanyan Wang, Tianle Chao, Qing Li, Peipei He, Lu Zhang, Jianmin Wang

**Affiliations:** 1 Shandong Provincial Key Laboratory of Animal Biotechnology and Disease Control and Prevention, College of Animal Science and Veterinary Medicine, Shandong Agricultural University, Tai’an 271000, China; 17826621820@163.com (Y.W.); chaotianle@sdau.edu.cn (T.C.); l18853852560@163.com (Q.L.); 2022110335@sdau.edu.cn (P.H.); ka205486665@163.com (L.Z.); 2 Key Laboratory of Efficient Utilization of Non-Grain Feed Resources (Co-Construction by Ministry and Province), Ministry of Agriculture and Rural Affairs, College of Animal Science and Veterinary Medicine, Shandong Agricultural University, Tai’an 271000, China

**Keywords:** metabolomics, transcriptomics, steroidogenesis, goat, ovarian development, sexual maturity

## Abstract

The ovary is a crucial reproductive organ in mammals, and its development directly influences an individual’s sexual maturity and reproductive capacity. To comprehensively describe ovarian sexual maturation in goats, we integrated phenotypic, hormonal, metabolomic, and transcriptomic data from four specific time points: after birth (D1), at 2 months old (M2), at 4 months old (M4), and at 6 month old (M6). The study showed that during the early stage (D1–M2), ovarian growth was the most rapid, with weight and morphology increasing by 284% and 65%, respectively, and hormone levels rose significantly, with estradiol increasing by 57%. Metabolomic analysis identified 1231 metabolites, primarily lipids, lipid molecules, and organic acids, which can support hormone balance and follicle development by providing energy and participating in signaling transduction. Transcriptomic analysis identified 543 stage-specific differentially expressed genes, mainly enriched in steroid biosynthesis, amino acid metabolism, and the PI3K/AKT pathway, which are key factors influencing ovarian cell proliferation, apoptosis, hormone secretion, and metabolism. The integrated analysis revealed the key processes in the ovarian steroid hormone biosynthesis pathway and gene/metabolite networks associated with ovarian phenotypes and hormone levels, ultimately highlighting scavenger receptor class B type 1 (*SCARB1*), Cytochrome P450 Family 1 Subfamily A Member 1 (*CYP11A1*), 3beta-hydroxysteroid dehydrogenase (*3BHSD*), progesterone, estradiol, and L-phenylalanine as key regulators of ovarian morphological and functional changes at different developmental stages. This study is the first to reveal the metabolic changes and molecular regulatory mechanisms during ovarian sexual maturation in goats, providing valuable insights for understanding reproductive system development and optimizing reproductive performance and breeding efficiency.

## 1. Introduction

Goats (*Capra hircus*) are important livestock in China, providing meat, skin, wool, and fiber products to the population [1]. Their reproductive traits [2,3], such as ovulation rate and litter size, are important for assessing their economic value. The ovary is the most important reproductive organ in female goats, mediating oocyte maturation, ovulation, and steroid hormone secretion, thereby significantly influencing their fecundity [4]. The Jining Gray (JG) goat, a superior local breed in Shandong Province, is renowned for its high reproductive rate, early sexual maturity (first ovulation at 2 months) [5,6], and year-round estrus, achieving sexual maturity significantly earlier than other goats (3–4 months) [7,8]. Therefore, JG goats display distinct ovarian function and hormonal regulation characteristics during early growth stages, offering an ideal model for studying ovarian development during sexual maturation and more possibilities.

Sexual maturity refers to the period in animals after birth when they have gone through puberty and achieved the ability for normal reproduction [9]. This process involves significant changes in the physiological status and functional characteristics of the ovaries, encompassing follicular development, oogenesis, and the secretion of gonadal hormones. At birth, the ovaries are small and largely inactive, in a dormant state. As puberty approaches, the gradual reduction in estradiol’s negative feedback on luteinizing hormone-releasing hormone (LHRH) secretion allows for changes in gonadotropin-releasing hormone (GnRH) and its regulated levels of follicle-stimulating hormone (FSH) and luteinizing hormone (LH), thereby stimulating ovarian development, increasing estrogen and progesterone (PROG) secretion, and ultimately leading to estrus and ovulation [10]. Subsequently, with the combined action of various reproductive hormones, the ovaries gradually mature, with rapid follicular development and a significant increase in ovulation frequency. The reproductive and endocrine functions of the ovaries reach a peak, supporting normal estrous cycles and marking the onset of sexual maturity [5]. It is evident that steroid hormones play a crucial role in ovarian development. They not only regulate follicular development and maturation but also influence key processes such as cell proliferation, apoptosis, tissue remodeling, metabolism, and immune regulation, thereby ensuring normal ovarian function [11,12,13]. However, the specific molecular mechanisms controlling the ovarian development of goats during sexual maturity remain unclear, particularly the influence of the steroid hormone biosynthesis pathway.

The primary aim of metabolomics is to identify and quantitatively analyze dynamic metabolites produced in living organisms under various physiological states, nutritional conditions, drug metabolism, and environmental factors [14]. Transcriptome analysis is a powerful method for studying the expression patterns of genes involved in metabolite biosynthesis. In recent years, the integration of these two methods has gained attention for its ability to effectively analyze the relationships between genes and metabolites and elucidate the molecular mechanisms underlying phenotypic changes [15,16,17]. Studies have shown that ovarian development is accompanied by significant changes in metabolic pathways (e.g., amino acid metabolism [18], glyceride metabolism [19], steroid hormone biosynthesis [20], fatty acid synthesis [21], and protein digestion and absorption) that are closely related to follicular development, cell proliferation, apoptosis, and reproductive cycle regulation, particularly those pathways involved in ovarian steroid hormone biosynthesis. For example, the expression of the enzymes *CYP17A1* and *HSD3B2* together promoted the synthesis of 17-hydroxyprogesterone, thereby facilitating the entire process of follicular development [13]. TGFα and FGF2 inhibited Aromatase in vitro and reduced the secretion of estradiol (E2) in vivo [22]. FSH primarily promoted the development of follicles in goats by preventing granulosa cell apoptosis, likely by increasing IGF-I and steroid production through the PKA pathway [23]. Meanwhile, IGF-I effectively stimulated follicle growth and estradiol secretion through its interaction with its receptor and binding proteins [24]. Retinoic acid enhanced ovarian steroidogenesis by regulating the *MESP2/STAR/CYP11A1* pathway [25]. Polyunsaturated fatty acids influenced the steroid production by bovine primary granulosa cells [26]. Based on appropriate lipid sources, it was beneficial for steroid hormone synthesis, which improved the fertilization rates and oocyte diameter [27].

However, no researchers have yet used transcriptomic and metabolomic techniques to study the molecular and metabolic mechanisms of ovarian development during sexual maturity in goats. This study integrated metabolomic and transcriptomic analyses, combined with statistical measurements of serum reproductive hormones and ovarian phenotypic indicators, to comprehensively characterize the metabolomic and transcriptomic characteristics of the ovaries at four developmental stages, and to explore the mechanisms of ovarian development and the regulatory network of steroid hormone biosynthesis. This research will provide important insights for further elucidating the regulatory mechanisms of metabolism and gene expression in the ovarian development of goats during sexual maturation, offering new perspectives on the functional adaptation of ovaries at different developmental stages.

## 2. Results

### 2.1. Changes in Ovarian Phenotypic Characteristics and Reproductive Hormone Levels during Sexual Maturation

**Figure 1:**
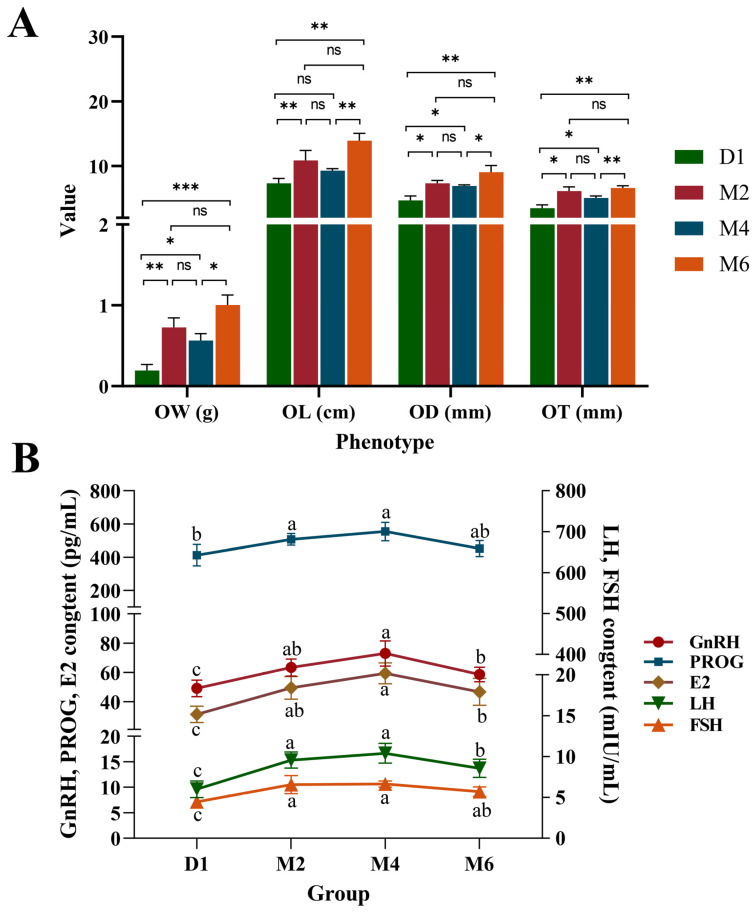
Analysis of ovarian phenotypic characteristics and serum hormone levels at four developmental stages. (**A**) Analysis of the ovarian weight (OW), ovarian length (OL), ovarian width (OD), and ovarian thickness (OT). (**B**) Levels of gonadotropin-releasing hormone (GnRH), luteinizing hormone (LH), follicle-stimulating hormone (FSH), progesterone (PROG) and estradiol (E2) in serum. D1: after birth, M2: at 2 months old, M4: at 4 months old, M6: at 6 months old. Values represent mean ± standard error. Different lowercase letters indicate significant differences (*p* < 0.05). * *p* < 0.05, ** *p* < 0.01, *** *p* < 0.001. ns = not statistically significant.

To understand the developmental changes in goat ovaries during sexual maturation, we measured ovarian phenotypic indicators and related reproductive hormone levels at four specific stages: after birth (D1), at 2 months old (M2), at 4 months old (M4), and at 6 months old (M6), and found significant intergroup differences in all indicators. From the D1 to M2 period, ovarian weight (OW), ovarian length (OL), ovarian width (OD), and ovarian thickness (OT) increased by 284%, 62%, 56%, and 76%, respectively. (Figure 1A). The ovarian weight in the M4 group was 0.56 ± 0.08 g, three times that at birth, but not significantly different from the M2 group, while significantly different from the M6 group. This indicated that the ovarian growth rate was faster in the early stages than in the later stages. Meanwhile, all hormone levels exhibited a trend of first increasing and then decreasing, with both them and the growth pattern of ovarian tissue showing phased characteristics during development (Figure 1B). From the D1 to M2 period, hormone levels significantly increased, while there was no significant change from the M2 to M4 period. This trend is consistent with the changes in ovarian phenotypes during these periods, indicating that changes in hormone levels may play an important role in ovarian development during sexual maturity.

### 2.2. Dynamic Metabolic Profiles of Ovaries during Sexual Maturation

**Figure 2:**
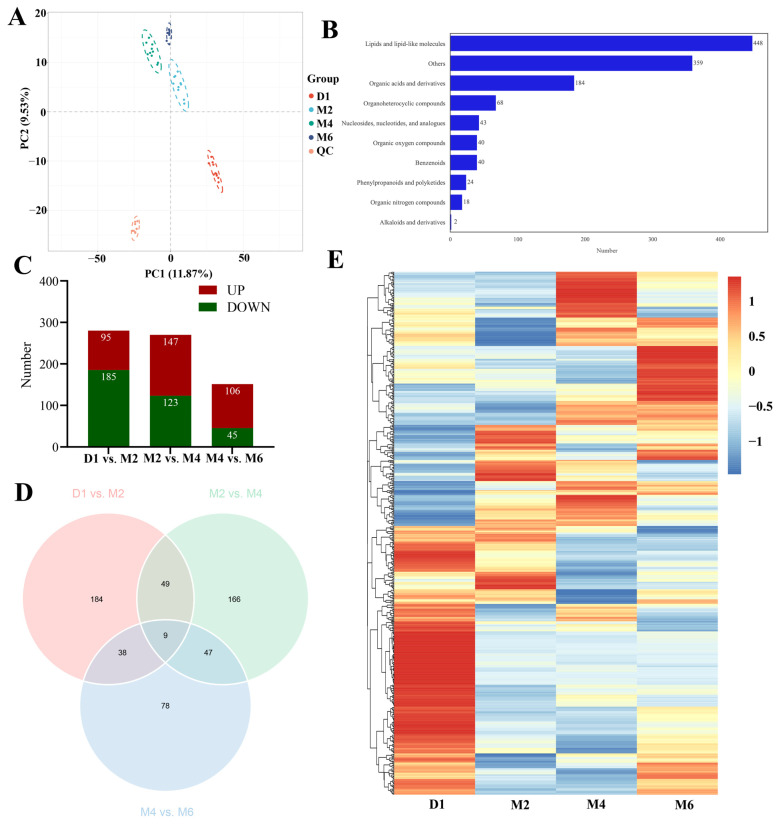
Basic metabolomics analysis of ovarian tissues at different developmental stages. (**A**) Principal component analysis of metabolites at four developmental stages. (**B**) Top 10 classes of metabolites identified during ovarian development. (**C**) The number of DAMs in pairwise comparisons. (**D**) The Venn diagram of DAMs in pairwise comparisons. (**E**) The heatmap of DAMs during ovarian development.

To better understand the metabolic changes in ovarian development during sexual maturity, we applied non-targeted metabolomics based on LC-MS/MS to analyze ovarian tissues at four specific developmental stages. Principal component analysis (Figure 2A) revealed significant differences in metabolite concentrations among the four groups, which was further confirmed by orthogonal partial least squares discriminant analysis (OPLS-DA) (Figure S1). After identification and analysis, 1231 metabolites were detected, which can be categorized into 10 major classes, including 448 lipids and lipid-like molecules, 184 organic acids and derivatives, 68 organoheterocyclic compounds, 359 others, etc. (Figure 2B, Table S2). In the three adjacent pairwise comparisons, we identified 570 differentially accumulated metabolites (DAMs) (Figure 2C and Figure S2), with the most found in the D1 vs. M2 comparison (95 upregulated, 185 downregulated) (Table S3). The top five DAMs with the most significant intergroup differences include Prostaglandin D2, 8-Hydroxyquinoline, 2-Hydroxyvaleric acid, ACar 15:2, and Indole-3-acetic acid. Furthermore, there were 9 common DAMs (Figure 2D), including steroids and steroid derivatives (methandrostenolone), fatty acyls (FAHFA (4:0/16:0) and dodecanedioic acid), carboxylic acids and derivatives (N-isovalerylglycine), and glycerophospholipids (LPS 16:1), detected across all comparison groups, suggesting that they may play key roles in certain functions or processes during ovarian development. The heatmap revealed significant differences in metabolite abundance across the four developmental stages, with about half of the DAMs highly expressed at the D1 stage and remaining low at the M2, M4, and M6 stages, while about one-sixth exhibited the opposite trend (Figure 2E).

**Figure 3:**
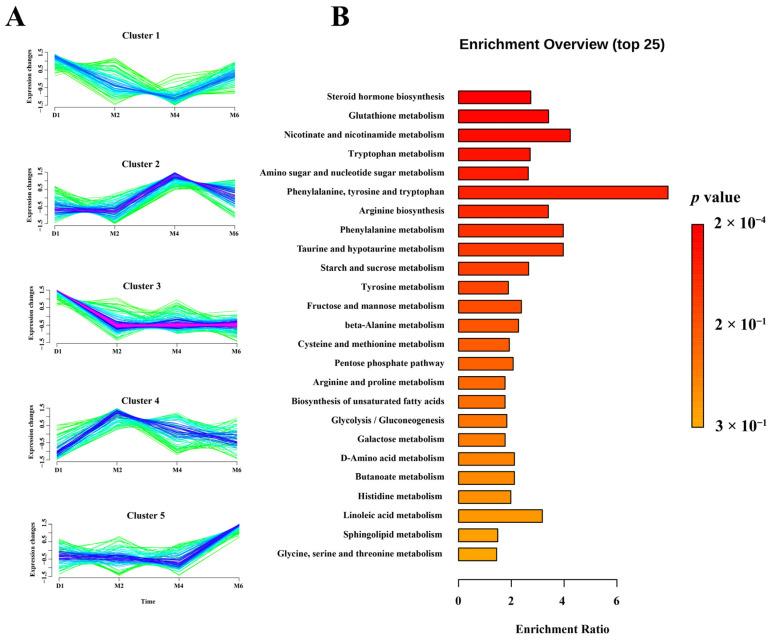
The expression patterns and functional enrichment analysis of DAMs. (**A**) Expression pattern analysis of DAMs. (**B**) KEGG pathway enrichment analysis of all DAMs.

To further investigate the trends and metabolic pathways of all DAMs, we performed clustering and Kyoto Encyclopedia of Genes and Genomes (KEGG) enrichment analysis on these metabolites (Figure 3A, Table S4). Cluster 1 DAMs (101), with glycerophospholipids (24) and carboxylic acids and derivatives (17) being the most abundant, had the highest levels during the D1 stage and the lowest during the M4 stage, and were significantly enriched in pathways such as amino acid metabolism (phenylalanine, tyrosine, and tryptophan biosynthesis; tyrosine metabolism; phenylalanine metabolism), signaling pathways (glucagon, AMPK), and carbon metabolism. Cluster 2 DAMs (123), with fatty Acyls being the most abundant, reached their highest levels at the M4 stage, primarily involving pathways related to taste and olfactory transduction, neuroactive ligand-receptor interaction, cGMP-PKG and FcεRI signaling, and metabolic regulation (steroid hormone biosynthesis, aldosterone synthesis and secretion, ovarian steroidogenesis, protein digestion and absorption). These results suggest that the ovaries at this stage are in a state of high metabolic and signaling activity, including enhanced sensory transduction, hormone synthesis, and energy metabolism, contributing to ovarian maturation and function. Cluster 3 DAMs (159), with amino acids, peptides, and analogs (27) being the most abundant, were at their highest levels in the D1 stage, enriched in pathways such as sphingolipid metabolism, sphingolipid signaling, glycine, serine and threonine metabolism, glycerolipid metabolism, steroid hormone biosynthesis, and the estrogen pathway. In Cluster 4 (92 DAMs), fatty acyls (23) were the most abundant, while in Cluster 5 (95 DAMs), glycerophospholipids (44) were the most abundant, with their levels peaking in the M2 and M6 stages, respectively. Cluster 4 was primarily enriched in phenylalanine metabolism, fatty acid elongation, and biosynthesis, while Cluster 5 was enriched in mineral absorption, 2-oxocarboxylic acid metabolism, protein digestion and absorption, and aminoacyl-tRNA biosynthesis. Additionally, we conducted KEGG enrichment analysis on all DAMs, revealing significant enrichment in pathways such as steroid hormone biosynthesis (with the highest significance and the largest number of metabolites), followed by glutathione metabolism, nicotinate and nicotinamide metabolism, tryptophan metabolism, amino sugar and nucleotide sugar metabolism, and Phenylalanine, tyrosine and tryptophan biosynthesis (Figure 3B, Table S4). These findings suggest that these pathways may be involved in the metabolic activities and biological function changes in the ovary at different developmental stages.

### 2.3. Dynamic Transcriptomic Profiles of Ovaries during Sexual Maturation

**Figure 4:**
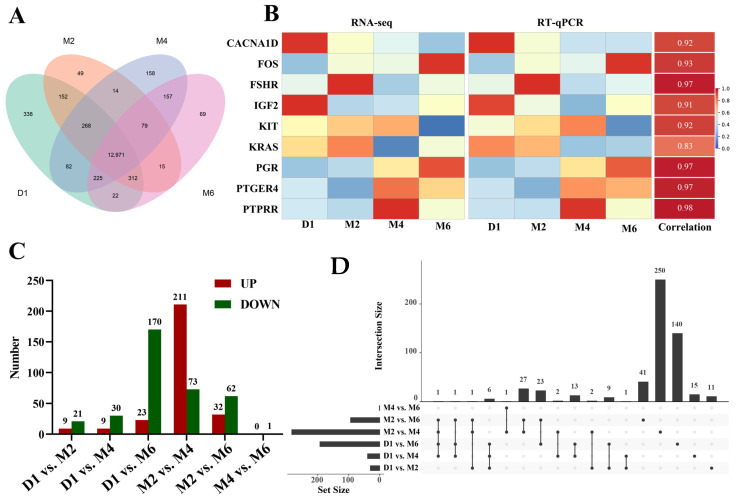
Basic transcriptome analysis of ovarian tissues at different developmental stages. (**A**) Venn diagram of the number of mRNA in ovary tissues at different developmental stages. (**B**) Validation of transcriptome data by qRT-PCR. (**C**) The number of DAMs in pairwise comparisons. (**D**) The Upset Plot for DEGs in different comparison groups.

**Figure 5:**
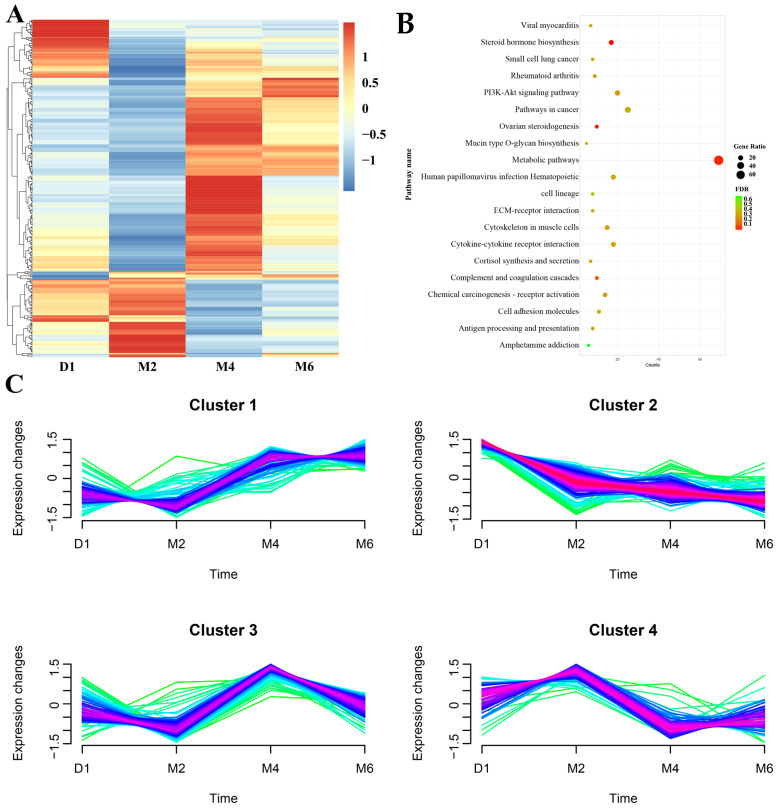
The expression patterns and functional enrichment analysis of DEGs. (**A**) The heatmap of DEGs during ovarian development. (**B**) KEGG pathway enrichment analysis of all DEGs. (**C**) Expression pattern analysis of DEGs.

A total of 258.6 Gb of clean reads were obtained from the 20 rRNA-depleted RNA-Seq libraries, with a sequencing error rate of 0.03%, Q20 and Q30 base percentages of 96.55% and 90.90%, respectively, and GC content ranging from 47.10% to 50.43%, indicating good sequencing quality. Over 94.64% of the reads were successfully mapped to the goat genome (Table S5). A total of 14,283 mRNAs (Fragments Per Kilobase of transcript per Million mapped reads, FPKM > 0.5) were identified, with 338, 49, 158, and 69 specifically expressed at stages D1, M2, M4, and M6, respectively (Figure 4A). To validate the RNA-seq data, 9 randomly selected differentially expressed genes (DEGs) were analyzed using real-time quantitative reverse-transcription PCR (RT-qPCR). The results were consistent with the sequencing data, with an average correlation of 0.93 (Figure 4B, Table S6), indicating that the RNA-seq data are reliable and suitable for further research. Among the six comparison groups, a total of 543 DEGs were identified, with the highest number of DEGs between the M2 and M4 stages (211 upregulated and 74 downregulated), including 250 specifically expressed DEGs (Figure 4C,D and Table S7), indicating more active gene expression during this stage. The DEG expression change heatmap reflects their stage-specific patterns during ovarian development (Figure 5A). 

To further investigate the expression patterns and potential functions of DEGs in the ovary during sexual maturation, we conducted expression clustering, as well as KEGG enrichment analyses. All DEGs were categorized into four clusters (Figure 5C, Table S8). Cluster 1 genes were primarily enriched in immune and disease-related pathways, such as pertussis and antigen processing and presentation. Cluster 2 genes were enriched in the cytoskeleton in muscle cells, GnRH secretion, and metabolic pathways. Cluster 4 genes exhibited high expression at the M2 stage and were significantly enriched in pathways such as PI3K-Akt signaling, prolactin signaling, steroid hormone biosynthesis, ovarian steroidogenesis, ECM–receptor interaction, and protein digestion and absorption. This indicates that cell proliferation, growth, hormone synthesis and regulation, and cellular activities in the ovaries were highly active during this stage, likely associated with the biological processes supporting the first estrous ovulation. Compared to the above-mentioned metabolite distribution patterns, Cluster 3 and metabolome Cluster 2 showed highly similar trends, both enriched in key metabolic pathways such as steroid hormone biosynthesis, ovarian steroidogenesis, and tyrosine metabolism. This suggests a potential regulatory relationship between gene transcription levels and metabolite distribution during ovarian development. Additionally, functional enrichment analysis of all DEGs revealed significant enrichment in pathways such as steroid hormone biosynthesis (with the highest significance), ovarian steroidogenesis, metabolic pathways, and PI3K-AKT signaling (Figure 5B, Table S8). This further indicates that the steroid hormone biosynthesis pathway plays a crucial role in the physiological state and reproductive capacity of the ovary during sexual maturation, and their specific mechanisms and functions are worth investigating.

### 2.4. Regulation of Gene Expression and Metabolites in the Ovaries during Sexual Maturation

**Figure 6:**
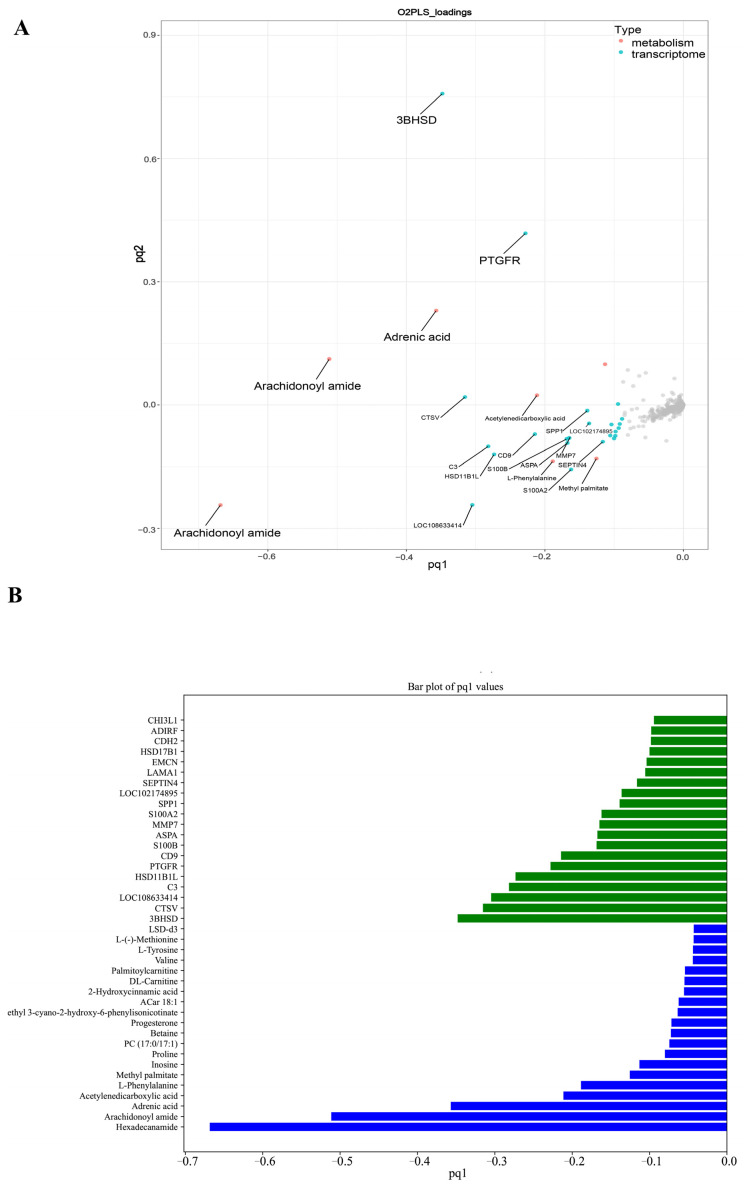
The integrated metabolomic and transcriptomic analysis of ovarian tissues at different developmental stages. (**A**) The O2PLS analysis of DAMs and DEGs. (**B**) Bar plot of the top 20 transcriptomics and metabolites pq1.

We conducted an integrated analysis of the transcriptome and metabolome to further understand the regulatory mechanisms underlying ovarian development during sexual maturation. Using the two-way orthogonal PLS (O2PLS) model with all DEGs and DAMs, we found that changes in the transcriptome significantly impact the metabolome (Figure 6A). The top 10 genes strongly influencing the metabolome included 3-beta-hydroxysteroid dehydrogenase (*3BHSD*), cathepsin V (*CTSV*), complement component 3 (*C3*), hydroxysteroid 11-beta dehydrogenase 1-like (*HSD11B1L*), and prostaglandin F receptor (*PTGFR*), with three genes (*3BHSD*, *HSD11B1L*, and hydroxysteroid 17-beta dehydrogenase 1 (*HSD17B1*)) involved in ovarian steroid hormone synthesis.

Additionally, we identified the top 10 metabolites significantly affecting the transcriptome, including Hexadecanamide, Arachidonoyl amide, Adrenic acid, Acetylenedicarboxylic acid, and L-Phenylalanine, with 5 belonging to Lipids and 4 to Carboxylic acids (Figure 6B). Furthermore, cluster analysis (Figure 3A and Figure 5C) revealed that metabolome Cluster 3 and transcriptome Cluster 2 showed highly similar trends, with both being highly expressed at the D1 stage. These metabolites and genes exhibited significant positive correlations (R > 0.8, *p* < 0.01) (Table S9). The top 10 metabolites with the highest significance, mainly belonging to phenylpropanoids and polyketides, followed by benzenoids, were significantly positively correlated with 43 genes (Figure S3), which were mainly enriched in metabolic pathways (Table S10). Metabolome Cluster 2 showed highly similar or opposite trends to transcriptome Clusters 3 and 4, respectively, peaking at the M4 stage or showing the lowest expression at M4, with significant correlations between these metabolites and genes (R > |0.5|, *p* < 0.01) (Tables S11 and S12). The top 10 most significant metabolites, primarily belonging to lipids and benzenoids, were significantly positively and negatively correlated with 202 genes (Figure S4), which were significantly enriched in steroid hormone biosynthesis, PI3K-Akt signaling, metabolic pathways, etc., (Table S10).

### 2.5. Gene Expression and Metabolite Changes in the Ovarian Steroid Hormone Biosynthesis Pathway

**Figure 7:**
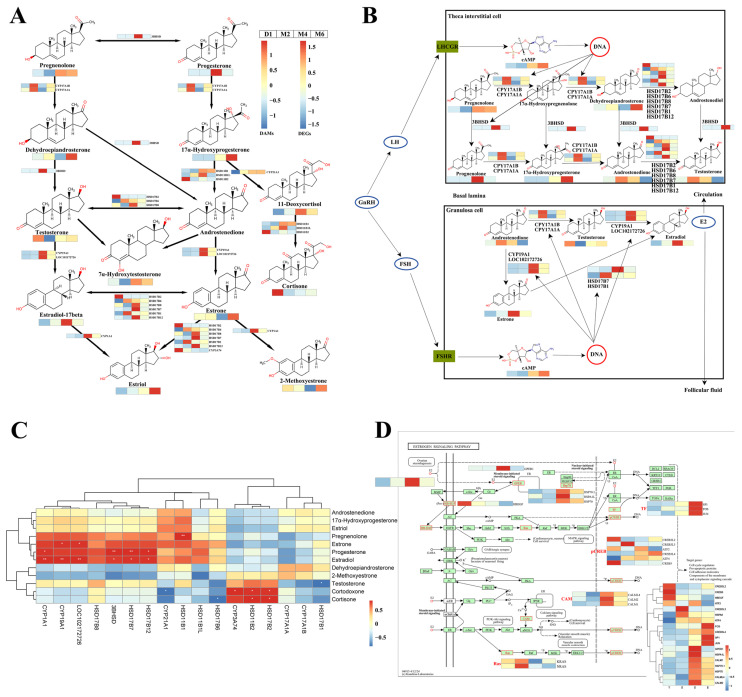
The pathway related to steroid hormone synthesis and the heatmap of changes in metabolites and genes. (**A**) The steroid biosynthesis pathway. (**B**) The ovarian steroid synthesis pathway. (**C**) Correlation analysis of DAMs and DEGs involved in steroid synthesis. Blue indicates a negative correlation and red indicates a positive correlation. The “*” and “**” represent significant differences at levels of *p* < 0.05, *p* < 0.01 according to Pearson’s correlation coefficient. (**D**) The estrogen signaling pathway during ovarian development.

Based on our research findings, we identified 17 DEGs and 13 DAMs in the steroid hormone biosynthesis pathway. As shown in Figure 7A,B, we examined the relationship between the expression levels of these DEGs and the abundance of key DAMs, observing significant changes across different developmental stages. The low expression levels of most genes involved in the regulation of steroidogenesis at birth may reflect a low requirement for steroid hormones in the early stages of life, indicating that the ovaries are not yet active. The pathway of ovarian steroid synthesis included the following key steps: cholesterol was converted into pregnenolone by the Cytochrome P450 Family 1 Subfamily A Member 1 (*CYP11A1*), then metabolized to progesterone (PROG) by *3BHSD*, and subsequently converted into androst-4-ene-3,17-dione (androstenedione) and 17beta-hydroxy-4-androsten-3-one (testosterone) by steroid17α-hydroxylase/17,20-lyase A (*CYP17A1A*) and 17α-hydroxylase/17,20-lyase B (*CYP17A1B*). Finally, androstenedione and testosterone were converted into estradiol through the actions of estradiol 17-beta-dehydrogenase (*HSD17B*) and steroid 19-alpha-hydroxylase (*CYP19A1*). Additionally, we observed that the expression patterns of most genes involved in the regulation of steroidogenesis differed from the trends in metabolite changes, indicating a potentially complex dynamic relationship between gene expression and metabolite regulation. However, the expression patterns of some genes, particularly those in the hydroxysteroid dehydrogenase family and the cytochrome P450 family, were consistent with changes in the metabolite, suggesting that these genes may play a more direct or critical role in the regulation of steroid hormone synthesis. *CYP1A1*, *CYP19A1*, and *LOC102172726* were genes involved in the late-stage regulation of estradiol synthesis. Their expression profiles increased gradually with age, peaking at the M4 stage, and showed a significant positive correlation with estradiol levels (*p* < 0.05), directly influencing estradiol synthesis (Figure 7C). The trends in estrone and estradiol were also highly consistent. In the steroid hormone synthesis pathway, they could interconvert through *HSD17B* (Figure 7A), suggesting the presence of such a pathway in the ovary. Pregnenolone, an intermediate in estradiol synthesis, was significantly positively correlated with the expression of the *3BHSD* gene, with both peaking at the M4 stage. *3BHSD*, *HSD17B7*, and *HSD17B12* were also significantly positively correlated with estradiol and progesterone (Figure 7C), and they are important enzymes in the synthesis of estradiol. Interestingly, the trend in estradiol levels was consistent with the changes in blood E2 levels. Estradiol synthesis was directly regulated by LH and FSH and indirectly by GnRH. GnRH, LH, FSH, PROG, and E2 in the serum also showed the same trend (Figure 1B). Consequently, significant changes in steroid hormone biosynthesis in ovarian tissues at different developmental stages revealed a correlation between gene expression profiles and metabolite variations. Additionally, the estrogen signaling pathway during ovarian development was analyzed, and 19 DEGs were detected, most of which were highly expressed in the M4 stage, consistent with changes in estrogen levels (Figure 7D). Estrogen, via receptors like G protein-coupled estrogen receptor 1 (*GPER1*), regulates key genes and pathways, influencing the cell cycle, apoptosis, cell adhesion, and signal transduction [28], ensuring normal ovarian function and structure during development.

### 2.6. Screening Key Metabolites Associated with Ovarian Phenotypes and Hormone Levels through Weighted Gene Co-Expression Network Analysis (WGCNA)

**Figure 8:**
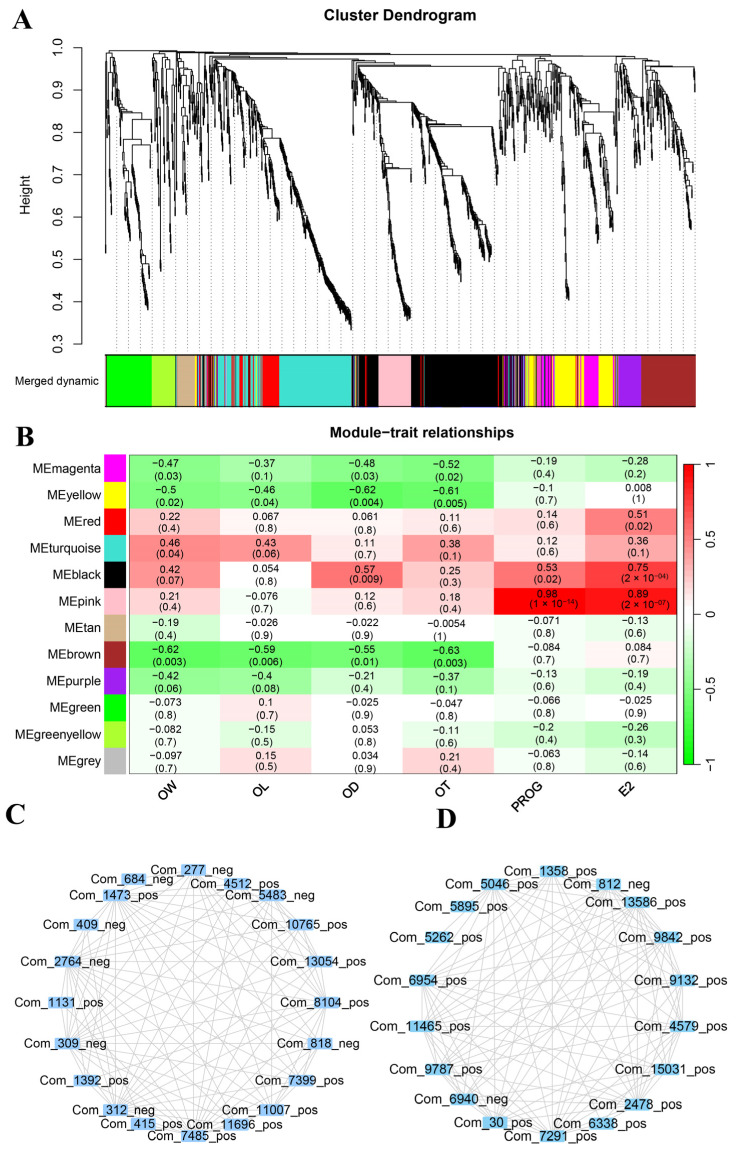
Analysis of the WGCNA of the ovarian metabolome at different developmental stages. (**A**) The clustering dendrogram of all metabolites. The color rows below the dendrogram show module assignments based on the dynamic hybrid branch cutting method. Each color represents a different module of co-expressed metabolites, while the “Merged dynamic” row indicates merged modules, with gray representing unassigned metabolites. The y-axis (Height) represents the dissimilarity between metabolites, with higher values indicating less similarity. (**B**) Heatmap of the correlations between modules and phenotypic traits. Modules are color-coded on the left, and phenotypic traits are listed below. The numbers in the cells represent correlation coefficients, with red for positive correlations and green for negative correlations. Color intensity indicates correlation strength. (**C**,**D**) Visualization of connections of metabolites in pink (**C**) and brown (**D**) modules. According to the connection weight value, only the first 100 functional relationships are shown in the figures. Yellow nodes represent hub metabolites with high connectivity, while blue nodes represent others. The thickness of the edges between nodes reflects the strength of their connections, with thicker edges indicating stronger associations, highlights key metabolites in the modules. OW: ovarian weight. OL: ovarian length. OD: ovarian width. OT: ovarian thickness. E2: estradiol. PROG: progesterone.

To investigate the potential regulatory network of metabolites during goat ovarian development, we constructed a WGCNA network for all metabolites and analyzed the dynamic features related to ovarian phenotypes and hormone level change (Table S13). The dynamic tree cutting identified 12 modules with similar expression patterns, each distinguished by a different color (Figure 8A), representing metabolite co-expression relationships (Figure 8A). In the module–trait relationship analysis (Figure 8B), the pink module was significantly positively correlated with serum E2 and PROG levels, with correlation coefficients of 0.98 and 0.89, respectively (*p* < 0.05). This module identified 73 metabolites (Table S14), including 19 lipids, 8 organic acids, and 8 organoheterocyclic compounds, with steroids and their derivatives being the most abundant, such as progesterone, estradiol, 5α-pregnan-3,20-dione, 7-ketocholesterol, hydroxyprogesterone caproate, etiocholanolone, and methyltestosterone. The brown module was significantly negatively correlated with OW, OL, OD, and OT, with correlation coefficients all below −0.55 (*p* < 0.05) (Figure 8B). This module identified 120 metabolites (Table S15), including 28 lipids (17 of which were fatty acyls) and 14 organic acids (10 of which were carboxylic acids), further highlighting the important influence of lipids and organic acids on ovarian development. Based on the connection weight values, we further visualized the networks of the top 100 functional relationships among metabolites in the pink and brown modules. Finally, in the pink module (Figure 8C, Table S16) and the brown module (Figure 8D, Table S17), seven key metabolites (e.g., progesterone, deoxyguanosine, and modafinil acid) and five key metabolites (e.g., cinnamic acid, D(+)-phenyllactic acid, and 3-phenyllactic acid) were identified, respectively, which are closely related to hormone regulation and ovarian morphogenesis.

### 2.7. Screening Key Genes Associated with the Ovarian Phenotype and Hormones Using WGCNA

**Figure 9:**
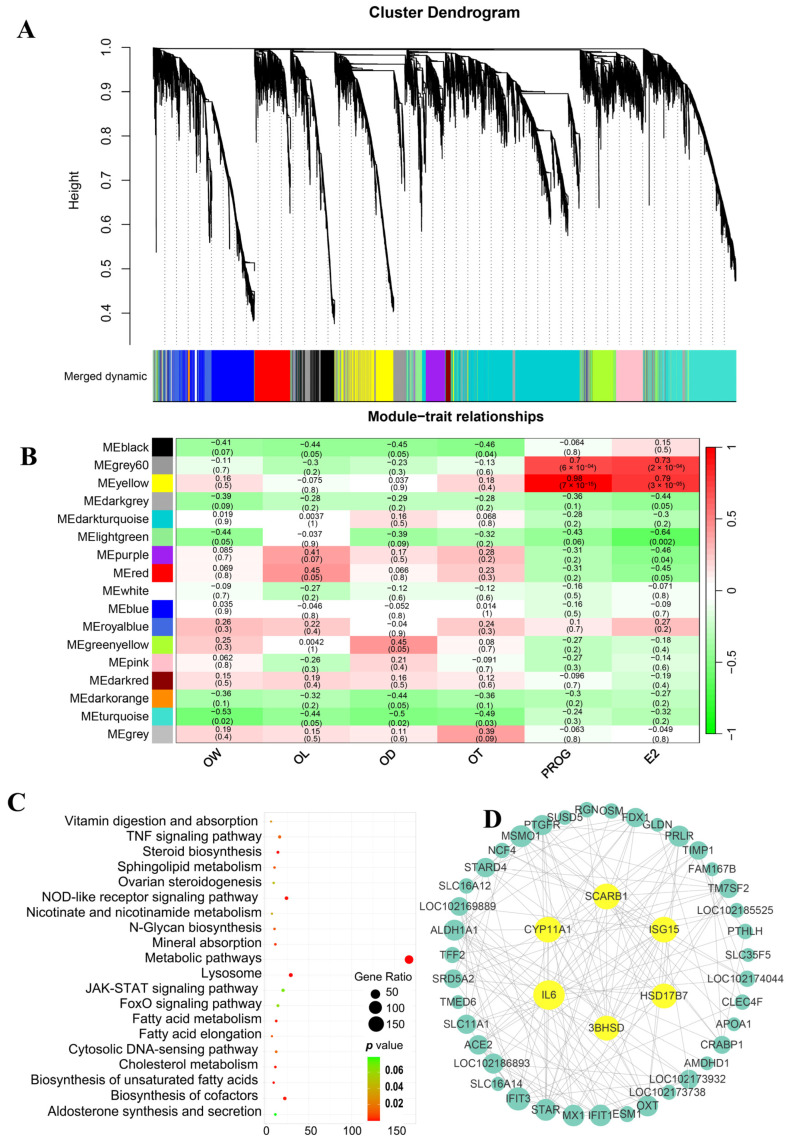
Analysis of the WGCNA of ovarian transcriptome at different developmental stages. (**A**) The clustering dendrogram of all genes. The color rows below the dendrogram show module assignments based on the dynamic hybrid branch cutting method. Each color represents a different module of co-expressed genes, while the “Merged dynamic” row indicates merged modules, with gray representing unassigned metabolites. The y-axis (Height) represents the dissimilarity between genes, with higher values indicating less similarity. (**B**) Heatmap of the correlations between modules and phenotypic traits. The modules are color-coded on the left, and phenotypic traits are listed below. The numbers in the cells represent correlation coefficients, with red for positive correlations and green for negative correlations. Color intensity indicates correlation strength. (**C**) KEGG enrichment analysis of genes in the yellow module. The y-axis shows enriched biological pathways, and the x-axis (Count) indicates the number of genes involved. (**D**) Network visualization of the top 50 genes in the yellow module, based on connection weights. Yellow nodes represent hub genes with high connectivity, while blue nodes represent other genes. Edge thickness reflects connection strength, with thicker edges indicating stronger associations. OL: ovarian length. OD: ovarian width. OT: ovarian thickness. E2: estradiol. PROG: progesterone.

The remaining genes after filtering out low-expression genes (FPKM < 1) were used for constructing the WGCNA. Seventeen distinct modules were identified, each represented by a different color, reflecting unique gene co-expression patterns (Figure 9A, Table S18). In the module–trait relationship analysis (Figure 9B), the turquoise module was negatively correlated with OW, OL, OD, and OT, while the yellow and gray 60 modules showed a positive correlation with serum E2 and PROG levels (correlation coefficient > 0.7, *p* < 0.05) (Figure 9B). To determine the potential functions of these modules, we performed KEGG pathway enrichment analysis on the genes of these three modules (Table S19). The results showed that the turquoise module was significantly enriched in the Basal transcription factors pathway. The yellow module was significantly enriched in 18 pathways, particularly those related to steroid biosynthesis, such as steroid biosynthesis, metabolic pathways, the biosynthesis of unsaturated fatty acids, and cholesterol metabolism (Figure 9C). The gray 60 module was primarily enriched in 17 pathways related to energy metabolism and diseases, including oxidative phosphorylation, propanoate metabolism, and non-alcoholic fatty liver disease. These findings emphasized the gene modules associated with ovarian development and hormone regulation, especially with the yellow module potentially playing a key role in ovarian morphogenesis and hormone regulation. Therefore, based on the kWithin values (representing gene connectivity within the module), we further screened 50 candidate hub genes from the yellow module (Table S20) and constructed a PPI network (Figure 9D). Ultimately, six potential hub genes closely related to ovarian development were identified: *SCARB1*, *CYP11A1*, *IL6*, *ISG15*, *HSD17B7*, and *3BHSD*.

## 3. Discussion

Sexual maturation is a critical developmental stage that animals undergo after birth, during which the ovaries experience significant morphological and functional changes influenced by specific hormones, growth factors, and their receptors [29,30]. Our study showed that significant differences in ovarian phenotypic characteristics and reproductive hormones at different developmental stages. The D1 stage had lower phenotypic indicators and hormone levels, indicating that the ovaries were not yet developed at this stage. At the M2 stage, the significant increase in hormone levels likely promoted rapid ovarian growth and development, leading to an increase in ovarian weight and volume, consistent with previous findings [5]. From the M2 stage to the M4 stage, there were no significant changes in hormone levels or ovarian morphology, suggesting that this period may be a phase of ovarian functional adjustment. From the M4 stage to the M6 stage, hormone levels decreased, but ovarian morphology changed. These findings suggested that the early developmental stages were characterized by rapid ovarian morphological growth, while after 60 days, the focus shifted to the improvement of ovarian function. The rapid growth and development of the ovaries in the early postnatal period might form the intrinsic basis for the high reproductive performance of JG goats.

Metabolomic analysis identified 1231 metabolites, with “lipids and lipid-like molecules” being the most abundant. This class of metabolites has been proven to play a crucial role in follicular development and ovarian hormone synthesis [31,32,33,34]. A total of 570 DAMs were identified, with the highest number found between the D1 and M2 stages. Clustering and KEGG enrichment analyses revealed that the metabolic profile of ovarian tissue is closely related to developmental stages. Metabolites exhibiting high levels at the D1 stage were mainly enriched in amino acid metabolism pathways (including phenylalanine, tyrosine, and tryptophan biosynthesis, as well as tyrosine metabolism) and signaling pathways (such as glucagon, AMPK, and sphingolipids). Amino acids, as fundamental components of proteins, play crucial roles in cell growth and repair [35]. Particularly in the early stages of ovarian development, high levels of amino acid synthesis and metabolism support rapid cell division and tissue growth. AMP-activated protein kinase (AMPK) is an energy state sensor that maintains cellular energy homeostasis, regulating not only metabolism but also cell growth and proliferation [36]. It has been reported that the ovaries of goats grow rapidly in morphology within 0–30 days after birth, with significant increases in weight and volume (five times higher than at birth) [5]. This is consistent with our experimental results, indicating that ovarian development during this period heavily relies on energy metabolism and amino acid metabolism to meet the demands of rapid growth and development. Metabolites exhibiting high levels at the M2 stage were highly enriched in lipid metabolism pathways, which play a key role in follicular development and oocyte maturation. Fatty acid oxidation serves as a vital energy source for oocytes, supporting their growth and maturation [21] while also influencing luteinizing hormone levels, which in turn affects ovarian development and function [37]. The presence of lipids, especially saturated and unsaturated fatty acids, can affect the lipid content and developmental competence of oocytes in in vitro maturation systems [31]. Metabolites exhibiting high levels at the M4 stage were highly enriched in pathways closely related to ovarian function, such as FoxO, PI3K-Akt [38], and mTOR [39,40] signaling, as well as steroid hormone biosynthesis [13,41,42], all of which play crucial roles in ovarian development, follicle activation, and reproductive cycle regulation. The metabolites with higher levels in the M6 stage were primarily involved in pathways related to energy metabolism, amino acid and fatty acid synthesis, and hormone synthesis, such as protein digestion and absorption, amino acid synthesis and prolactin signaling, indicating an increased demand for energy and hormones in the ovaries during this period. Therefore, our results indicated that the M2 and M4 stages were significant periods of ovarian functional maturation and structural adjustment, aligning with the early sexual maturity characteristic [7,8] of JG goats, which exhibited unique ovarian functions and hormone regulation during early growth. We also performed transcriptome analysis and identified a total of 14,283 expressed genes, with 543 genes showing differential expression. Clustering and enrichment analyses revealed that these genes exhibit significant temporal and spatial specificity at different stages. They are mainly enriched in biological processes and signaling pathways related to cell proliferation, apoptosis, hormone secretion, and metabolism, such as steroid hormone biosynthesis, ovarian steroidogenesis [20], and PI3K-Akt [43], GnRH, oxytocin and metabolic pathways. These findings are consistent with the annotation results of DAMs, further revealing the complex network of metabolic and gene expression regulation during ovarian development, with significant differences in metabolic pathways and regulatory mechanisms at different stages.

Through the integrated analysis of transcriptomic and metabolomic data, it was found that changes in the transcriptome significantly affect the metabolome, particularly in the ovarian steroid hormone biosynthesis pathway. The synthesis of steroid hormones is crucial for ovarian development, directly impacting follicle maturation, cell proliferation, apoptosis, and the regulation of the reproductive cycle [44]. This process requires the involvement of multiple enzymes and involves the conversion of cholesterol in endocrine glands into sex steroids [45], with estradiol typically being the final product, ensuring the normal function and adaptability of the ovaries at different developmental stages. In this study, 13 DAMs and 17 DEGs in the steroid hormone biosynthesis pathway were identified, showing significant changes across different developmental stages. Except for *HSD17B2*, *CYP3A74*, and *HSD11B2*, other genes involved in steroidogenesis regulation were significantly upregulated during the M2 and M4 stages compared to the other two stages, likely reflecting accelerated ovarian development and increased sex hormone synthesis during these periods. In the schematic diagram of the estradiol synthesis pathway, we found that pregnenolone was primarily metabolized by *3BHSD* into PROG, then converted by *CYP17A1A* and *CYP17A1B* into androstenedione and testosterone, and finally transformed into estradiol by *HSD17B* and *CYP19A1*. Notably, genes from the hydroxysteroid dehydrogenase family and cytochrome P450 family (e.g., *HSD17B8*, *HSD17B7*, *HSD17B12*, *HSD11B1L*, *HSD3B*, *CYP19A1*, and *CYP1A1*) exhibited expression patterns similar to the levels of PROG, E2, and estrone, peaking at the M4 stage. This suggested that these genes may play crucial regulatory roles in ovarian development, especially during key stages of sexual maturation. Studies have shown that *HSD17B7*, *HSD17B8*, and *HSD17B12* selectively catalyze the conversion between estrogen estrone and estradiol [46,47,48], though the involvement of *HSD17B12* in E2 production remains controversial. *HSD3B* supports progesterone synthesis [49], while the *CYP19A1* gene is associated with aromatase deficiency, a key enzyme in estrogen synthesis, whose deficiency leads to low estrogen and high androgen levels [50]. Our correlation analysis revealed that the expression trends of *3BHSD*, *HSD17B7*, and *HSD17B12* genes are significantly positively correlated with PROG, with all three showing high expression at the M4 stage. Regulatory genes involved in the later stages of steroid synthesis, such as *CYP19A1*, *LOC102172726*, and *HSD17B7*, are significantly positively correlated with estradiol levels, directly affecting its synthesis. Interestingly, the trend in estradiol levels mirrored the changes in blood E2 levels, with estradiol synthesis directly regulated by LH and FSH, and indirectly by GnRH. Additionally, the trends in blood GnRH, LH, FSH, PROG, and E2 levels were consistent. This further indicates that at different stages of ovarian development, the coordination between metabolite levels and gene expression follows a synchronized pattern. For example, the upregulation of metabolism and gene expression at the M2 stage signifies rapid ovarian development and increased hormone synthesis, while the high expression at the M4 stage corresponds to the functional peak of the ovaries during sexual maturity.

Differential analysis often overlooks key genes with subtle expression changes [51]. Therefore, we integrated all transcriptomic and metabolomic datasets and used WGCNA to generate gene–metabolite networks. By modeling specific metabolic pathways and examining co-expression patterns between ovarian phenotypes/hormones and individual genes/metabolites, we identified critical hub genes and metabolites potentially related to ovarian development. The results showed that the metabolites in the brown module and the genes in the turquoise module were closely related to the weight, length, width, and thickness of the ovaries, suggesting that these modules may play important roles in regulating ovarian morphology and function. The brown module identified 120 DAMs, primarily classified as lipids and organic acids, which play key roles in cell membrane structure, signal transduction, energy storage [52], and cellular metabolism [53], including cinnamic acid, D(+)-phenyllactic acid, and 3-phenyllactic acid. Additionally, we found that the metabolites in the pink module and the genes in the yellow module were positively correlated with serum E2 and PROG. In the pink module, the most abundant were steroids and their derivatives, such as progesterone, estradiol, 5α-pregnane-3,20-dione, and etiocholanolone, which play key roles in regulating follicle development, apoptosis, and the reproductive cycle [20,54]. Genes in the yellow module were significantly enriched in pathways related to steroid synthesis, metabolic pathways, the biosynthesis of unsaturated fatty acids, and cholesterol metabolism. This further confirms that changes in serum sex hormones are highly related to the steroid levels in the ovaries. We identified seven key metabolites (e.g., progesterone, deoxyguanosine, and modafinil acid) and six hub genes (*SCARB1*, *CYP11A1*, *IL6*, *ISG15*, *HSD17B7*, and *3BHSD*). Among these, *SCARB1*, *CYP11A1*, and *HSD3B* are crucial for maintaining ovarian cholesterol balance, luteal steroidogenesis, and follicle development [55,56,57], while *HSD17B7* is involved in the later stages of steroid hormone conversion [58]. However, the role of IL-6 signaling in regulating E2 expression in goat granulosa cells requires further investigation. These findings indicated that the key metabolites and hub genes identified, particularly those involved in steroid hormone synthesis, were important for regulating ovarian maturation, follicle development, and hormone levels.

However, the study has some limitations. Despite obtaining reliable data through a balanced experimental design and appropriate replication, the limited sample size may limit the statistical significance and generalizability of the results. We selected goats from the same farm, maintained consistent feeding conditions and management, and implemented strict experimental controls. However, potential confounding factors such as environmental conditions and measurement errors may still affect the interpretation of the results. Additionally, we filtered out some less expressed transcripts, which may have led to overlooking certain transcription factors and non-coding RNAs that, despite their low expression levels, play important roles in gene regulation [59]. Therefore, future studies could consider further increasing the population sample size and lowering the filtering threshold to include less expressed transcripts, thereby maximizing the reliability and generalizability of the results. Additionally, the identified potential key genes and metabolites can be further investigated through functional validation experiments to better understand their specific roles and interaction mechanisms in ovarian development. In conclusion, this study is the first to reveal key regulatory mechanisms of ovarian development during sexual maturation in goats by integrating ovarian phenotypes, hormone levels, metabolomics, and transcriptomic data. The study showed that during the early stage (D1–M2), ovarian growth was the most rapid, with weight and morphology increasing by 284% and 65%, respectively, and hormone levels rising significantly, with estradiol increasing by 57%. Metabolomics analysis identified 570 DAMs, with the highest abundance found in lipids, lipid-like molecules, and organic acids, which can support hormonal balance and follicle development through amino acid metabolism, steroid hormone biosynthesis, and energy metabolism. Transcriptomic analysis identified 543 stage-specific DEGs, mainly enriched in steroid biosynthesis, amino acid metabolism and the PI3K/AKT pathway, which are key factors influencing ovarian cell proliferation, apoptosis, hormone secretion, and metabolism. The integrated analysis revealed that the expression changes of DEGs significantly affected the levels of lipids, organic acids, and polyketides in the ovaries. Additionally, the analysis uncovered the key processes in the ovarian steroid hormone biosynthesis pathway and gene/metabolite networks associated with ovarian phenotypes and hormone levels, ultimately highlighting *SCARB1*, *CYP11A1*, *3BHSD*, progesterone, estradiol, and L-phenylalanine as key regulators of ovarian morphological and functional changes at different developmental stages. This study reveals the metabolic changes and underlying molecular regulatory mechanisms during ovarian sexual maturation in goats, providing potential molecular targets and fresh light for understanding reproductive system development, as well as optimizing reproductive performance and breeding efficiency. Additionally, the methods and findings presented may offer valuable insights for related studies of other species.

## 4. Materials and Methods

### 4.1. Ethics Statement

Animal experiments were conducted with the consent and guidance of the Animal Care and Use Committee of Shandong Agricultural University (SDAUA-2023–157).

### 4.2. Animal Materials and Tissue Sample Collection

In this study, 20 healthy female JG goats were obtained from the Jining Gray Goat Breeding Farm in Jiaxiang, Shandong Province, China. They were raised and managed under consistent conditions, with a diet consisting of a balanced mix of hay and concentrate, free access to food and water, and housing in well-ventilated sheds with ample space for movement. The goats were divided into four age groups: D1, M2, M4, and M6, with five goats per group. Specific information on the goats can be found in our previous study [60]. Each goat was slaughtered on a designated date, with the ipsilateral ovarian tissue surgically removed, measured for OW, OL, OD, and OT, then immediately snap-frozen in liquid nitrogen and stored at −80 °C until RNA extraction for NGS library construction and metabolite extraction.

### 4.3. Blood Sample Collection and Measurement of Reproductive Hormone Levels

Blood samples (10 mL) were drawn from the jugular vein of each experimental goat, left to stand for one hour, and then centrifuged at 3000× *g* for 10 min. The supernatant was transferred to RNase-free tubes and stored in liquid nitrogen for preservation. Serum hormone levels, including GnRH, FSH, LH, PROG, and E2, were measured using high-quality ELISA kits (MDBio, Qingdao, China) via the double-antibody sandwich method [61,62]. In this method, the first antibody is coated on the ELISA plate to capture the specific hormone from the serum sample. After washing to remove unbound substances, a second, enzyme-linked antibody specific to the hormone is added, which binds to the captured hormone. A substrate solution is then added, which reacts with the enzyme to produce a color change. The intensity of the color, measured as optical density (OD) at a wavelength of 450 nm, is directly proportional to the concentration of the hormone in the sample. Hormone levels were quantified using a standard curve generated from known concentrations of the hormones. Each sample was measured in triplicate, and the results were compared and evaluated using the mean values and their standard deviations.

### 4.4. Metabolomics Analysis

Following the previously reported method [63], the tissues (100 mg) were initially ground in liquid nitrogen before being resuspended in a pre-chilled 80% methanol solvent by well vortex. Non-targeted metabolomic analysis was conducted on 20 ovarian tissue samples from four developmental stages. The tissue (100 mg) was ground in liquid nitrogen, then suspended in a pre-cooled 80% methanol solution and vortexed. After maintaining on ice for 5 min and centrifuging at 15,000× *g* for 20 min, the supernatant was diluted to 53% methanol and injected into the LC-MS/MS system for analysis. The analysis was conducted using a Thermo Scientific Q ExactiveTM HF-X (Thermo Fisher Scientific, Waltham, MA, USA) mass spectrometer paired with a Vanquish UHPLC system, with the following parameters: a spray voltage of 3.5 kV, a capillary temperature of 320 °C, a sheath gas flow rate of 35 psi, an auxiliary gas flow rate of 10 L/min, an S-lens RF level of 60, and an auxiliary gas heater temperature of 350 °C.Analysis was performed using a C18 column (40 °C, flow rate of 0.2 mL/min, run time of 17 min) in both positive and negative ion modes. Data processing was conducted using Compound Discoverer 3.1 software for peak alignment, peak picking, and metabolite quantitation, with identification using mzCloud, mzVault, and MassList databases. The biological functional annotation of metabolites utilized the KEGG (https://www.genome.jp/kegg/, accessed on 15 June 2024), LIPID MAPS (www.lipidmaps.org, accessed on 15 June 2024), and the Human Metabolome Database (HMDB; http://www.hmdb.ca, accessed on 17 June 2024). We treated the samples of the same age group as duplicates and compared samples of different ages (i.e., D1 vs. M2, M2 vs. M4, and M4 vs. M6). The criteria for selecting DAMs were the variable importance in projection > 1, a *p* value < 0.05, and a fold change > 1.5 or <0.67. The *p*-values of metabolites between the two groups were evaluated using T-tests. Volcano plots and cluster heatmaps were generated using the ggplot2 [64] and pheatmap (https://CRAN.R-project.org/package=pheatmap, accessed on 17 June 2024) packages in R (v4.4.0), while time clustering analysis was performed using the mfuzz [65] package. Additionally, metabolic pathway enrichment analysis of differential metabolites was conducted using the KEGG database. The raw metabolomic data can be accessed on the MetaboLights database under the accession number MTBLS979433.

### 4.5. RNA Extraction, Quality Analysis and Illumina Sequencing

Total RNA was extracted from 20 ovarian tissue samples using TRIzol (Invitrogen, Carlsbad, CA, USA) following the manufacturer’s standard protocol. Briefly, 50–100 mg of tissue samples were homogenized in TRIzol, followed by phase separation with 300 μL of chloroform (J.T. Baker, Radnor, PA, USA). The aqueous phase containing RNA was transferred to a fresh tube containing 500 μL of isopropanol (J.T. Baker, Radnor, PA, USA) to precipitate the RNA. The RNA pellet was washed with 1 mL of 75% ethanol (J.T. Baker, Radnor, PA, USA), air-dried, and then dissolved in RNase-free water. The concentration, purity, and integrity of the RNA were measured using a NanoDrop 2000 spectrophotometer (Thermo Scientific, Wilmington, DE, USA) and an Agilent 5400 bioanalyzer (Agilent, Santa Clara, CA, USA). Higher RIN values generally indicate better RNA integrity, with RIN values ≥ 8 considered to be optimal for RNA analysis. RNA libraries were constructed using samples with RNA Integrity Number (RIN) values greater than 8. After removing rRNA from the RNA samples with the Ribo-Zero™ kit, the RNA were fragmented using a fragmentation buffer, reverse transcribed into cDNA with random primers, followed by double-stranded cDNA synthesis, purification, end repair, A-tailing, sequencing adapter ligation, USER enzyme degradation of the second strand, PCR enrichment, and AMPure XP beads purification to obtain the final strand-specific library. Suitable libraries were sequenced on the Illumina NovaSeq 6000 platform (Illumina, San Diego, CA, USA) using the PE150 (paired-end 150 bp) strategy.

Raw data in a fastq format were first processed using fastp (v0.19.4) [66]. In this step, clean data were obtained by trimming reads containing adapters, poly-N, or low-quality reads from the raw data. The goat reference genome (https://www.ncbi.nlm.nih.gov/datasets/genome/GCF_001704415.2/, accessed on 26 June 2024) and annotation files were downloaded from the NCBI database, and the reference genome index was built using Hisat2 (v2.0.5) [67], followed by aligning the paired-end clean reads to the reference genome. Transcript assembly and quantification were performed using StringTie (v2.2.3) [68]. Since paired-end sequencing was used in rRNA-depleted RNA-seq, transcript abundance was normalized to FPKM to facilitate accurate quantification with RSEM (v1.3.3) [69]. Differential expression analysis was performed using DESeq2 (v1.4.5) software [70]. We treated the samples of the same age group as duplicates and compared samples of different ages (i.e., D1 vs. M2, D1 vs. M4, D1 vs. M6, M2 vs. M4, M2 vs. M6, and M4 vs. M6). The Mfuzz (v2.64.0) software was used to cluster the DEGs using the c-means method. Gene Ontology (GO) annotation and KEGG pathway enrichment analysis of the selected DEGs were performed using the clusterProfiler (v4.12.6) tool [71], with terms or pathways considered significantly enriched if the *p*-value < 0.05. The raw RNA-seq files have been submitted to the NCBI Sequence Read Archive (SRA) under the project number PRJNA109117331. 

### 4.6. Integrated Analysis of Metabolomics and Transcriptomics

DAMs and DEGs were integrated to investigate the mechanisms of goat ovarian development. DAMs and DEGs were synchronously mapped onto KEGG pathway diagrams. Correlation analysis was conducted for the detected genes and metabolites in each sample group. The Pearson correlation coefficients between genes and metabolites were calculated using the COR program in R, and correlations with a PCC greater than 0.6 were represented in network diagrams. The O2PLS model was constructed using all DEGs [72], and analyzed with the o2m function from the OmicsPLS package [73]. High-correlation and high-weight DAMs and variables were identified through loading plots to filter out those that could significantly impact other global analyses. O2PLS is designed to handle complex interactions in multi-omic data, effectively capturing collinear structures between datasets while filtering out irrelevant noise, thereby improving the accuracy of data interpretation and model robustness. 

### 4.7. Weighted Gene Co-Expression Network Analysis 

To further screen for genes and metabolites closely related to ovarian development and steroid hormone synthesis, the WGCNA package [74] in R was used to perform weighted co-expression analysis on transcripts and metabolites, calculate the weighted expression correlation of genes and metabolites, and conduct hierarchical clustering analysis to construct gene and metabolite co-expression networks. Genes and metabolites with highly correlated expression were identified as a gene module, represented by the branches and different colors of the dendrogram. Then, correlation analysis was conducted between gene and metabolite modules and phenotypes and hormones to screen for modules related to yam tuber development. Parameter settings included the following: β = 7, CutHeight = 0.8, minSize = 30, with the rest set to default. Finally, the STRING (v12.0) [75] protein–protein association network was used and visualized with Cytoscape (v3.10.2) [76]. 

### 4.8. Real-Time Quantitative Reverse-Transcription PCR

To ensure the stability of RNA sequencing, 10 genes were randomly selected for RT-qPCR. Primers (Table S2) were designed using Beacon Designer and validated for specificity with NCBI Primer-BLAST. Total RNA was isolated from ovarian tissue using the TRIzol method. The Evo M-MLV RT Mix Kit with gDNA Clean for qPCR Ver.2 (Code: AG11728) was used to synthesize the first-strand cDNA of mRNA, and the SYBR Green Premix Pro Taq HS qPCR Kit (Code: AG11701) was used for the quantitative analysis of mRNA expression. RT-qPCR was performed on the LightCycler 480 instrument (Roche). Relative gene expression levels were calculated using the 2^−ΔΔCt^ method [77], with GAPDH as the internal reference (Table S1).

## Data Availability

The data presented in this study are available on request.

## Supplementary Materials

The following supporting information can be downloaded at: https://www.mdpi.com/article/10.3390/ijms25189898/s1.

## Abbreviations

JG	Jining Gray
OW	Ovarian weight
OL	Ovarian length
OD	Ovarian width
OT	Ovarian thickness
GnRH	Gonadotropin-releasing hormone
LH	Luteinizing hormone
LHRH	Luteinizing hormone-releasing hormone
LHCGR	Luteinizing hormone/choriogonadotropin receptor
FSH	Follicle-stimulating hormone
E2	Estradiol
PROG	Progesterone
OD	Optical density
RIN	RNA integrity number
PE150	Paired-end 150 bp
FPKM	Fragments per kilobase of transcript per million mapped reads
DAMs	Differential accumulated metabolites
DEGs	Differentially expressed genes
GO	Gene Ontology
KEGG	Kyoto Encyclopedia of Genes and Genomes
HMDB	Human Metabolome Database
OPLS-DA	Orthogonal partial least squares discriminant analysis
O2PLS	Two-Way orthogonal PLS
WGCNA	Weighted Gene Co-Expression Network Analysis
RTqPCR	Real-time quantitative reverse-transcription PCR
