# Supplementary figures and images for: Metabolomic and Transcriptomic Analyses Reveal the Potential Mechanisms of Dynamic Ovarian Development in Goats during Sexual Maturation
Source: Int J Mol Sci. 2024 Sep 13;25(18):9898. doi: 10.3390/ijms25189898 (PMC11432265; doi:10.3390/ijms25189898)

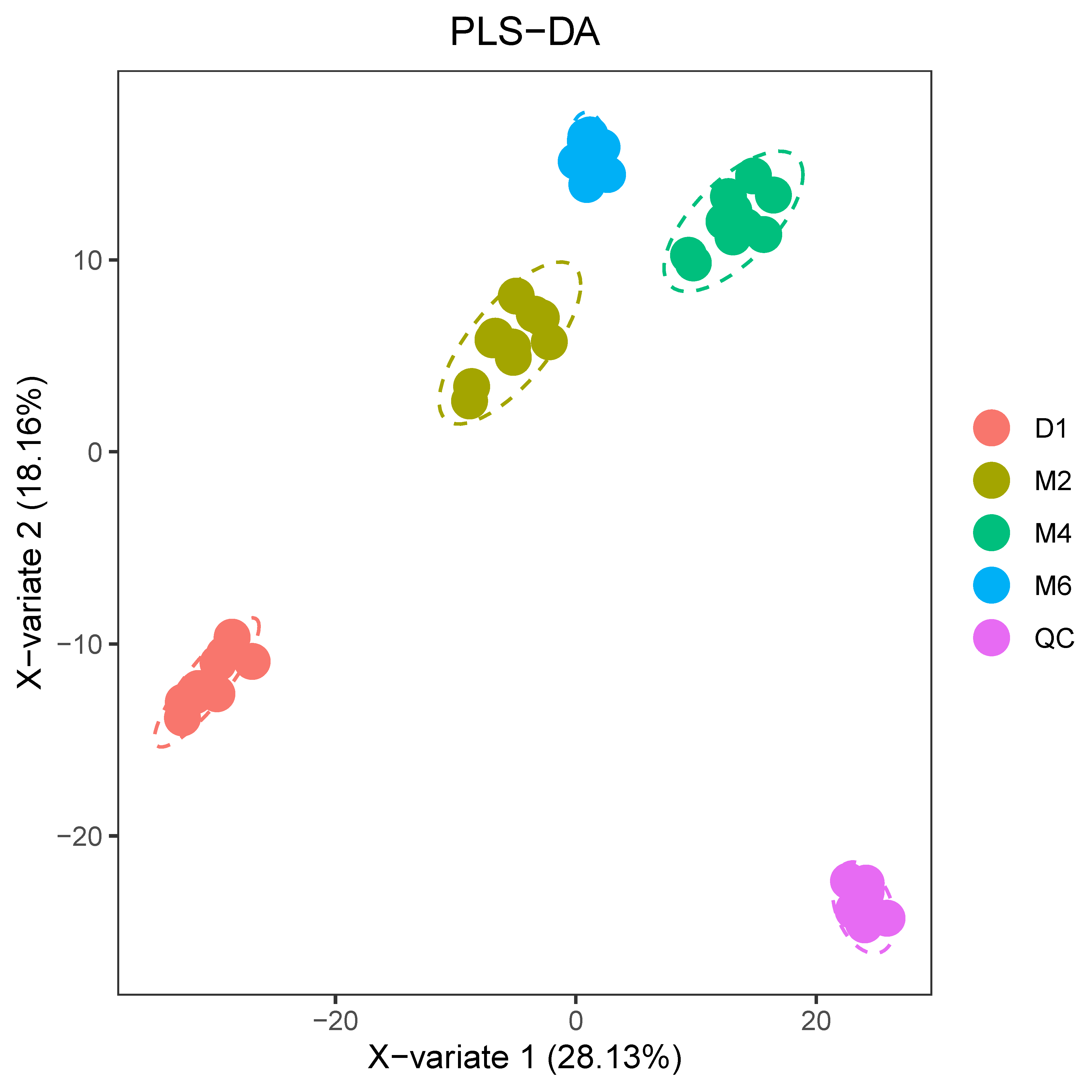

Supplement: Supplementary file 1 [file ijms-25-09898-s001.zip › Fig. S1.tif]

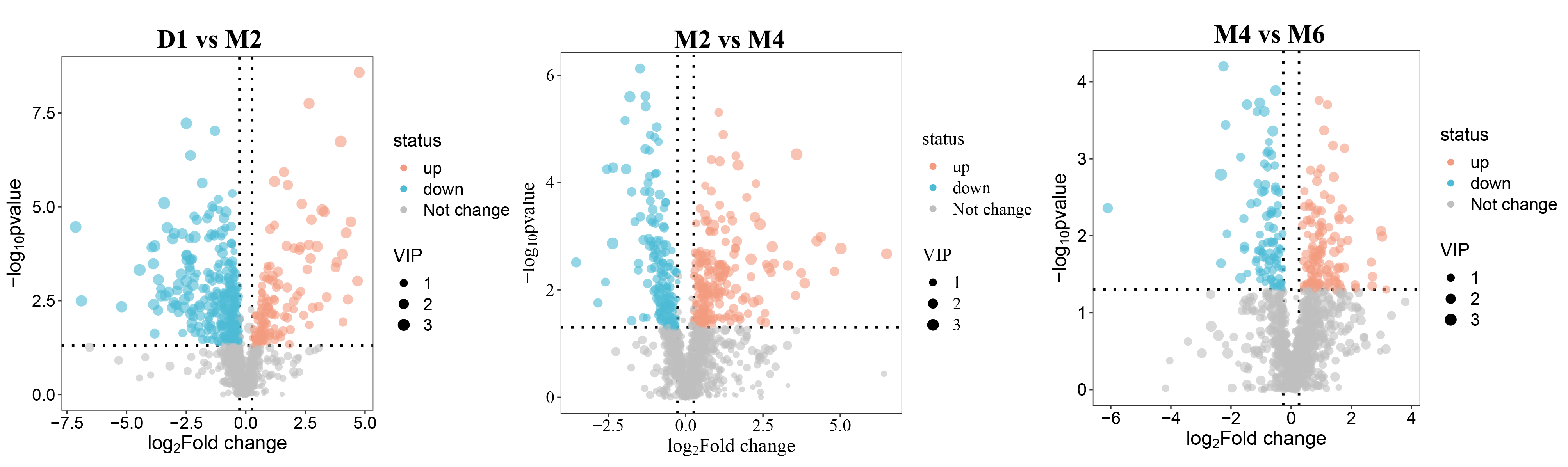

Supplement: Supplementary file 1 [file ijms-25-09898-s001.zip › Fig. S2.tif]

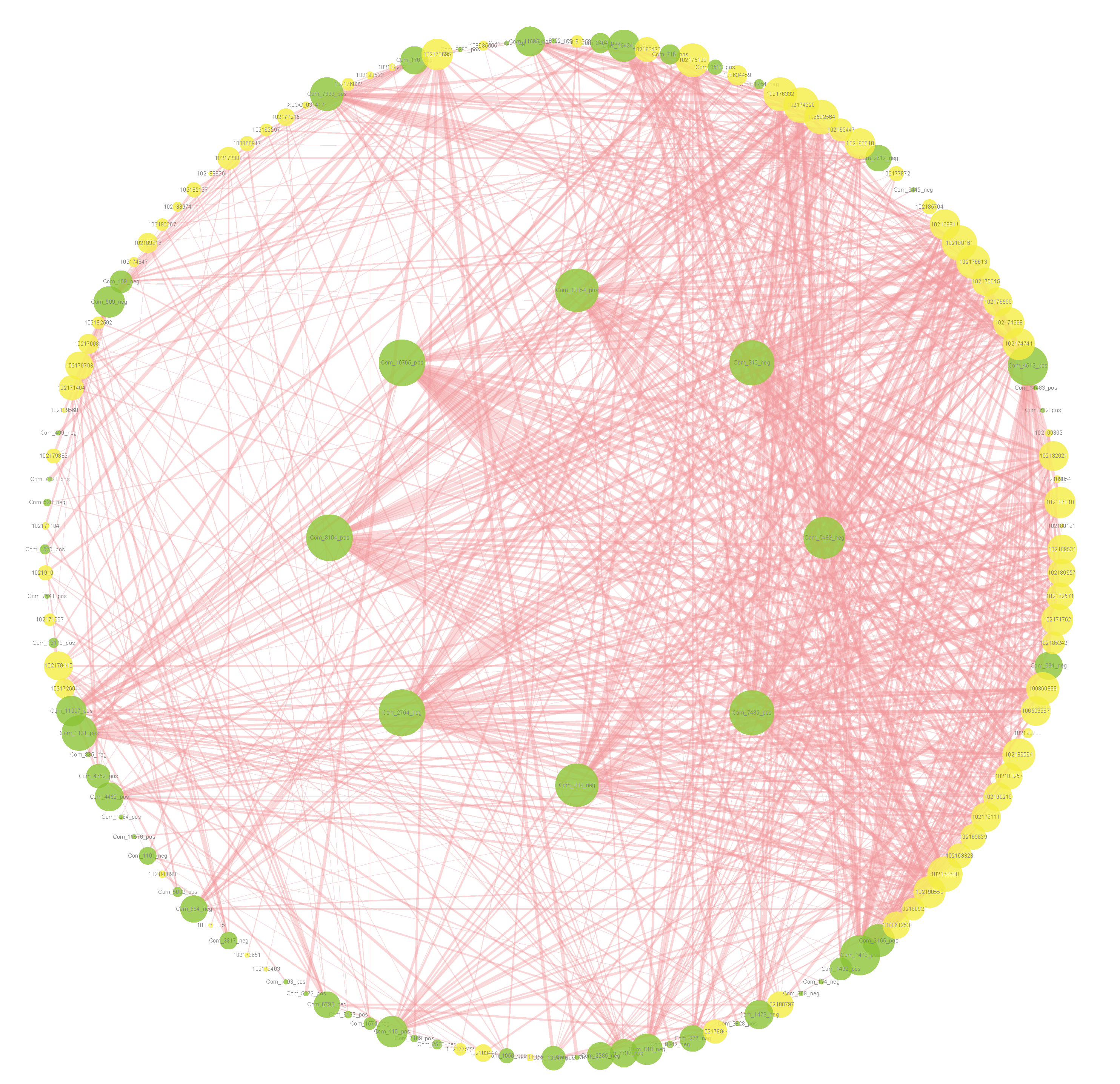

Supplement: Supplementary file 1 [file ijms-25-09898-s001.zip › Fig. S3.tif]

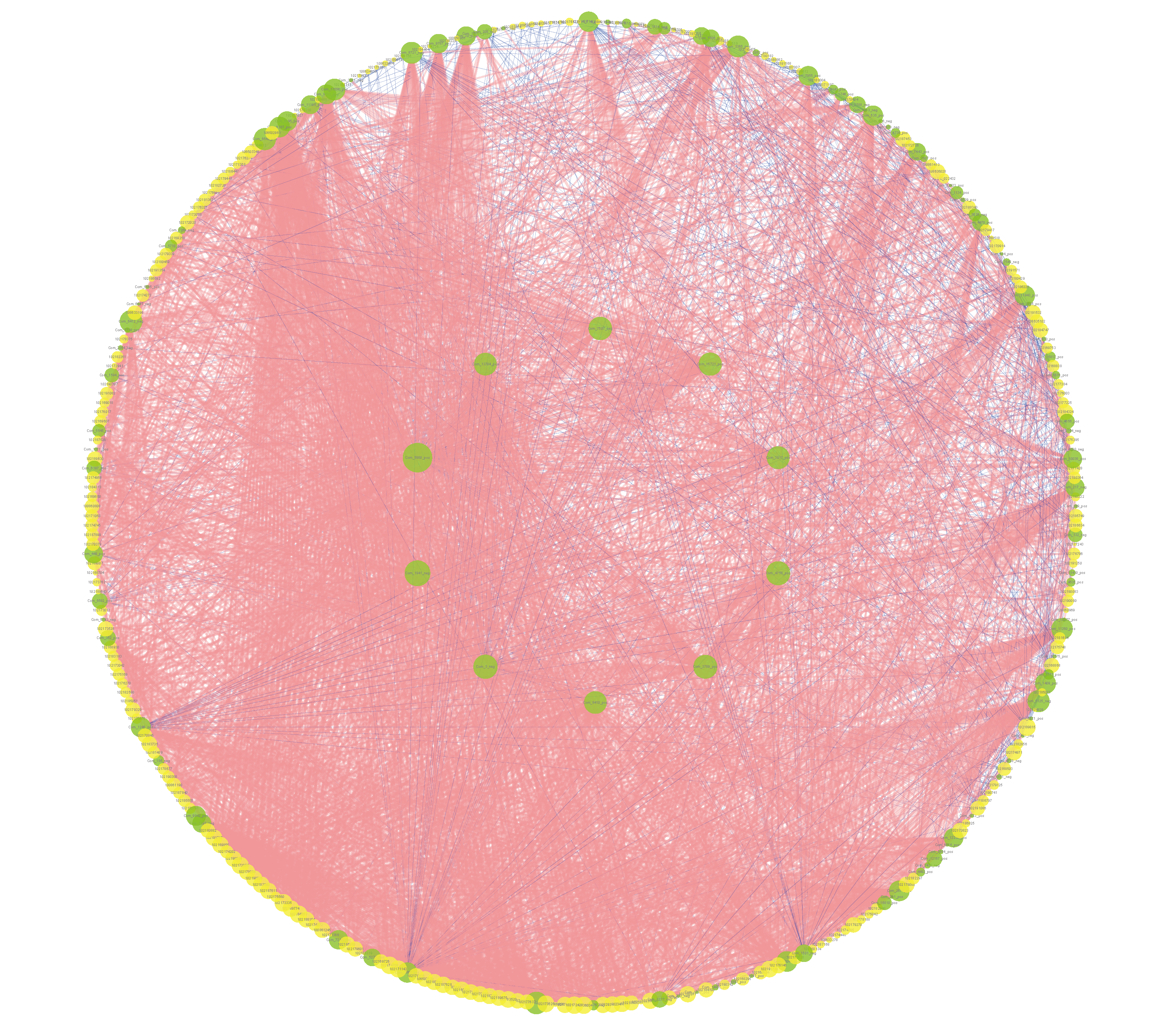

Supplement: Supplementary file 1 [file ijms-25-09898-s001.zip › Fig. S4.tif]
